# EPHA5 mutation was associated with adverse outcome of atezolizumab treatment in late-stage non-small cell lung cancers

**DOI:** 10.1186/s12890-022-02161-1

**Published:** 2022-09-19

**Authors:** Zhenxiang Li, Qing Zhou, Qi Wang, Haiyong Wang, Weiming Yue

**Affiliations:** 1 Medical Integration and Practice Center, Shandong University, Jinan, China; 2 Hospital of Traditional Chinese Medicine of Liaocheng City, Liaocheng, China; 3 Shandong Cancer Hospital and Institute, Shandong First Medical University and Shandong Academy of Medical Sciences, Jinan, China; 4 Department of Medical Oncology, Shandong Cancer Hospital and Institute, Shandong First Medical University and Shandong Academy of Medical Sciences, Jinan, 250117 China; 5 Department of Thoracic Surgery, Qilu Hospital of Shandong University, Jinan, 250012 Shandong China

**Keywords:** Lung cancer, Non-small cell lung cancer, Immunology, Biomarkers

## Abstract

**Background:**

The aim of the study was to investigate predictive value of gene mutation for atezolizumab treatment response from OAK and POPLAR cohorts.

**Methods:**

Several public databases were used for analyzing gene mutation type of EPHA5 and association with alterations of other genes. Survival analysis was performed for patients receiving atezolizumab from OAK and POPLAR cohorts.

**Results:**

EPHA5 mutation have high frequency to harbor TP53 and KEAP1 mutations. The bTMB value has significant difference between EPHA5 mutant and wild-type cases. Patients with EPHA5 mutation got worse survival compared to those without gene mutations receiving atezolizumab (*P* = 0.0186).

**Conclusions:**

EPHA5 mutant NSCLC may represent a subpopulation which showed worse response after treatment of atezolizumab compared to wild-type ones.

**Supplementary Information:**

The online version contains supplementary material available at 10.1186/s12890-022-02161-1.

## Background

Immunotherapy, based on targeting the programmed death-ligand 1(PD-L1)/PD-1, has been recently used to treat advanced non-small cell lung cancer (NSCLC) patients with no alteration in driver genes [1]. In both first- and second-line treatment settings, immunotherapy has been widely used either in combination with chemotherapy or as monotherapy in selected patients with high PD-L1 expressions [2–5]. Atezolizumab, an engineered humanized IgG1 monoclonal anti-PD-L1 antibody, can target PD-1 or PD-L1 for the treatment of NSCLCs. Unlike other agents that target the PD-L1/PD-1 axis, atezolizumab can block the interactions of PD-L1/PD-1 and PD-L1/B7.1, thereby preserving the immune homeostasis of normal tissues [6].

OAK and POPLAR, two randomized controlled clinical trials, compared the efficacy and safety of atezolizumab and docetaxel in patients with locally advanced or metastatic NSCLCs after the progression of post-platinum chemotherapy [6, 7]. Both the studies showed the beneficial effects of atezolizumab as compared to docetaxel in patients with NSCLCs and showed that the improvement in survival was also correlated with the PD-L1 expression. The two studies confirmed the predictive potential of the PD-L1 expression in response to atezolizumab treatment. However, in the OAK clinical trial, about 40% of patients with positive PD-L1 expression showed the disease progression after atezolizumab treatment [7]. Based on this perspective, clinical studies should be performed to explore the biomarkers, which are associated with the lack of response to atezolizumab treatment, and select appropriate patients for receiving immunotherapy.

Ephrin receptor A5 (EPHA5) belongs to Eph/ephrin family and plays an important role in the carcinogenesis of multiple cancers by binding to the ligand [8]. In several types of human cancers, including NSCLC, mutations in the EPHA5 gene have been found [8, 9]. A strong correlation between the mutations in EPHA5 and tumor immunity has been reported in studies. For example, EPHA5 mutation could impair natural killer (NK) cell-mediated cytotoxicity against the NSCLC cells [10]. A couple of studies showed that the lung adenocarcinoma patients with EPHA5 mutations from a Memorial Sloan-Kettering Cancer Center (MSKCC) cohort treated with immunotherapy exhibited significantly prolonged overall survival (OS) times, indicating that EPHA5 mutation might serve as a biomarker to predict the immune response of lung adenocarcinomas [11, 12]. The scenario was attributed to various mechanisms including correlation of EPHA5 mutations with enhanced tumor infiltrating lymphocytes (TILs), increased tumor mutational burden (TMB) and so on. However, the treatments of patients involved in the studies were complex, including anti-PD-1, anti-PD-L1, or combination treatments, which might lead to differences in the patient's responses.

This study analyzed the EPHA5 mutation types and their correlation with other gene mutations in NSCLC patients. Then, the predictive potential of EPHA5 mutations for atezolizumab treatment response in a second-line setting of the patients with previously treated NSCLCs was examined.

## Methods

### EPHA5 gene mutations in NSCLC

The type and frequency of gene mutations in the *EPHA5* gene were analyzed using cBioPortal database (https://www.cbioportal.org) [13, 14]. A total of 1668 samples were obtained from 1567 patients in the MSKCC cohort, who were diagnosed with NSCLC, for further analysis. Missense mutations and truncation in EPHA5 (The NCBI accession number: AB209375) were studied. Truncating mutations included nonsense, nonstop, frameshift deletion, frameshift insertion, and splice site mutations across the whole structure of the gene including transmembrane (TM) region. Detailed information of mutation was shown in Additional file 1: Table S1. The correlations of mutations in EPHA5 mutations with those in other genes were analyzed using mutual exclusivity (ME) analysis.

### Correlations of EPHA5 mutations with PD-L1 expression and blood TMB (bTMB) in OAK and POPLAR cohorts

The correlations of blood-based tumor mutational burden (bTMB) and PD-L1 expression with EPHA5 mutations, and their effects on prognosis were further evaluated in the OAK and POPLAR cohort. The data, relating to the expression of PD-L1 and bTMB in the OAK and POPLAR cohorts, have been published previously [7, 15]. Biomarkers mean PDL1 expression, bTMB data and EPHA5 mutation. Patients who could not provide information of these biomarkers were excluded from the study. In addition, patients with the synonymous gene mutations were not included shown in Additional file 2: Table S2. PDL1 expression was assessed by VENTANA SP142 PD-L1 immunohistochemistry assay (Ventana Medical Systems, Tucson, AZ, USA) [7]. PD-L1 expression was scored in tumor cells (as percentage of PD-L1-expressing tumor cells) and tumor-infiltrating immune cells (as percentage of tumor area) [6, 7]. The expression score criterion was as following: TC3 ≥ 50% and IC3 ≥ 10%, TC2 5%–50% and IC2 5–10%, TC1 1–5% and IC1 1–5% and TC0 < 1% and IC0 < 1%. TC3 was defined as strongly positive, while TC1/2/3 were defined as positive. The bTMB assay was conducted by counting all single-nucleotide variants at a variant allele fraction of at least 0.5% across 394 genes and estimating the tumor fraction according to maximum somatic allele frequency, filtering out germline events, and excluding driver mutations to generate a score. The unit of Y-axis (bTMB) in Fig. 2B was mutations per megabase. Subsequently, a total of 853 patients were included; their clinicopathological characteristics are listed in Table 1.

**Table 1:** Clinical variables of 853 patients between EPHA5 mutant and wild-type ones

Variables	*EPHA5* mutation (%)	None (%)	*P*
Total	65 (100)	788 (100)	
Age			0.544
< 65	38 (58.5)	430 (54.6)	
≥ 65	27 (41.5)	358 (45.4)	
Race			0.521
White	43 (66.2)	573 (72.7)	
Asian	15 (23.1)	149 (18.9)	
Others	7 (10.8)	66 (8.4)	
Sex			0.121
Female	19 (29.2)	307 (39.0)	
Male	46 (70.8)	481 (61.0)	
Histology			0.493
Squamous	17 (26.2)	238 (30.2)	
Non-squamous	48 (73.8)	550 (69.8)	
ECOG			0.319
0	18 (27.7)	266 (33.8)	
1	47 (72.3)	522 (66.2)	
Previous therapy			0.346
First-line chemotherapy	51 (78.5)	576 (73.1)	
Second-line chemotherapy	14 (21.5)	212 (26.9)	
Smoking			0.227
Current	13 (20.0)	125 (15.9)	
Never	6 (9.2)	134 (17.0)	
Previous	46 (70.8)	529 (67.1)	
Metastasis sites			0.004
≤ 3	34 (52.3)	547 (69.4)	
> 3	31 (47.7)	241 (30.6)	
Therapy			0.859
Atezolizumab	32 (49.2)	397 (50.4)	
Docetaxel	33 (50.8)	9.6)	

Annotation: *ECOG*, eastern cooperative oncology group. 0, fully active, able to carry on all pre-disease performance without restriction;1, means restricted in physically strenuous activity but ambulatory and able to carry out work of a light or sedentary nature

### Survival analysis

The correlations between EPHA5 mutations and OS of patients, receiving atezolizumab treatment, in the OAK and POPLAR cohorts were evaluated by performing a survival analysis. The differences in the survival of the patients, receiving atezolizumab and docetaxel treatments stratified with EPHA5 gene mutations and metastatic sites, were also evaluated.

### Ethical conduct

The current study was based on the previously published data; therefore, there was no personal identification of patients, and did not require informed consent.

### Statistical analyses

The differences in clinical variables of patients with EPHA5 mutations and wild-type EPHA5 were analyzed using chi-square. The difference of PDL1 expression between EPHA5 mutant and wild-type groups was evaluated by chi-square test. A *t*-test was used to examine the differences in blood-based tumor mutational burden (bTMB) between the patients having EPHA5 mutations and wild-type EPHA5. Kaplan–Meier analysis and log-rank test were used to plot and compare the survival curves, respectively. Multivariable COX regression model was used to calculate Hazard Ratio (HR) value (95%CI). The variables included were gene mutation status and metastatic sites number respectively. The data was shown in forest plot. SPSS 22.0 was used to perform the statistical analyses. All the tests were two-tailed, and a *P*-value < 0.05 was considered statistically significant.

## Results

### EPHA5 mutation types and their correlations with other genes mutations and patients’ variables

**Fig. 1:**
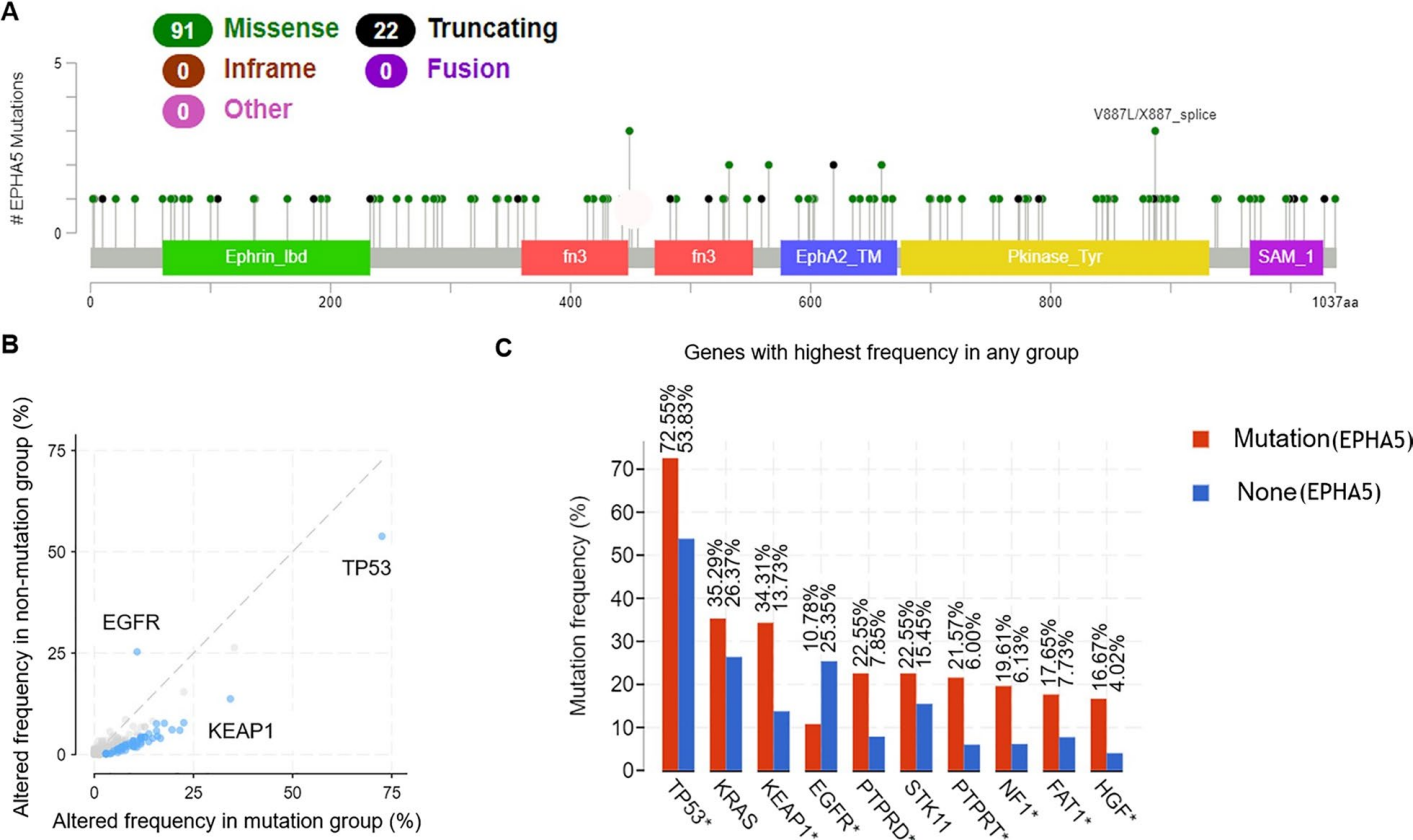
cBioPortal database was used for analyzing EPHA5 mutation type. Mutation diagram circles are colored with respect to the corresponding mutation types. In case of different mutation types at a single position, color of the circle is determined with respect to the most frequent mutation type (**A**). Mutual exclusivity and co-occurrence analysis. X-axis indicated that the altered frequency of genes in EPHA5 mutation patients and Y-axis revealed that in wild-type EPHA5 ones (the blue dot represents the specific gene mutation with significant *P* value) (**B**). The mutation frequency of related genes in the EPHA5 mutant and wild-type groups (* represents the difference meaningful) (**C**)

The types of mutations in the EPHA5 gene were determined using cBio Cancer Genomics Portal (MSK-IMPACT Clinical Sequencing Cohort). The missense and truncating mutations were the most frequently occurred mutations; the results were consistent with those reported previously, as shown in Fig. 1A and Additional file 1: Table S1 [9, 11]. 7.2% (113/1567) NSCLC patients carried EPHA5 mutations from MSKCC cohort. the major type of mutations was missense (91/113) whereas 22 patients harbored truncating mutations including Nonsense (8 patients), frameshift deletion (8 patients), splice site (5 patients) and frameshift insertion (1 patient). Among 113 patients, most of patients (92 patients) had adenocarcinoma while 10 patients diagnosed with squamous cell carcinoma. Others included large cell carcinoma (5 patients), non-small cell lung carcinoma (5 patients) and spindle cell carcinoma (1 patient). The co-occurrence of mutations in EPHA5 and other genes was also found in NSCLCs. Briefly, the patients with EPHA5 mutations had a high frequency to have co-mutations in tumor suppressor p53 (TP53) and Kelch-like ECH-associated protein 1 (KEAP1)*,* whereas patients with wild-type EPHA5 had mutations in Epidermal growth factor receptor (EGFR), as shown in Fig. 1B. The frequency of mutations in TP53, KEAP1*,* and EGFR between the patients with EPHA5 mutations and wild-type EPHA5 were 72.55% and 53.83%, 34.31% and 13.73%, and 10.78% and 25.35%, respectively (*P* < 0.05). Co-mutations in EPHA5 also existed with the genes protein tyrosine phosphatase, receptor type D (PTPRD), protein tyrosine phosphatase receptor T (PTPRT), Neurofibromatosis type 1 (NF1), FAT atypical cadherin 1 (FAT1), and hepatocyte growth factor (HGF) as shown in Fig. 1C.

A total of 853 patients in the OAK and POPLAR cohorts were enrolled in this study for further analysis. Among them, 527 patients (61.8%) were male, 713 patients (83.6%) had a history of smoking, 598 patients (70.1%) had the major histology as non-squamous, 627 patients (73.5%) were given first-line therapy before treating them with atezolizumab, 65 patients (7.62%) had mutations in EPHA5 gene*.* The clinical characteristics of the patients are listed in Table 1. The variables included in this study were age, race, sex, histology, Eastern Cooperative Oncology Group (ECOG) performance score, previous treatments (first -line or second -line chemotherapy), smoking history, and therapy regimen of the patients. These variables were not statistically significantly correlated with EPHA5 gene mutations. It was found that the mutation frequency of EPHA5 was more in the patients with more metastatic sites as compared to those with fewer metastatic sites (*P* = 0.004).

### Correlations of EPHA5 mutations with PD-L1 expression and bTMB level

**Fig. 2:**
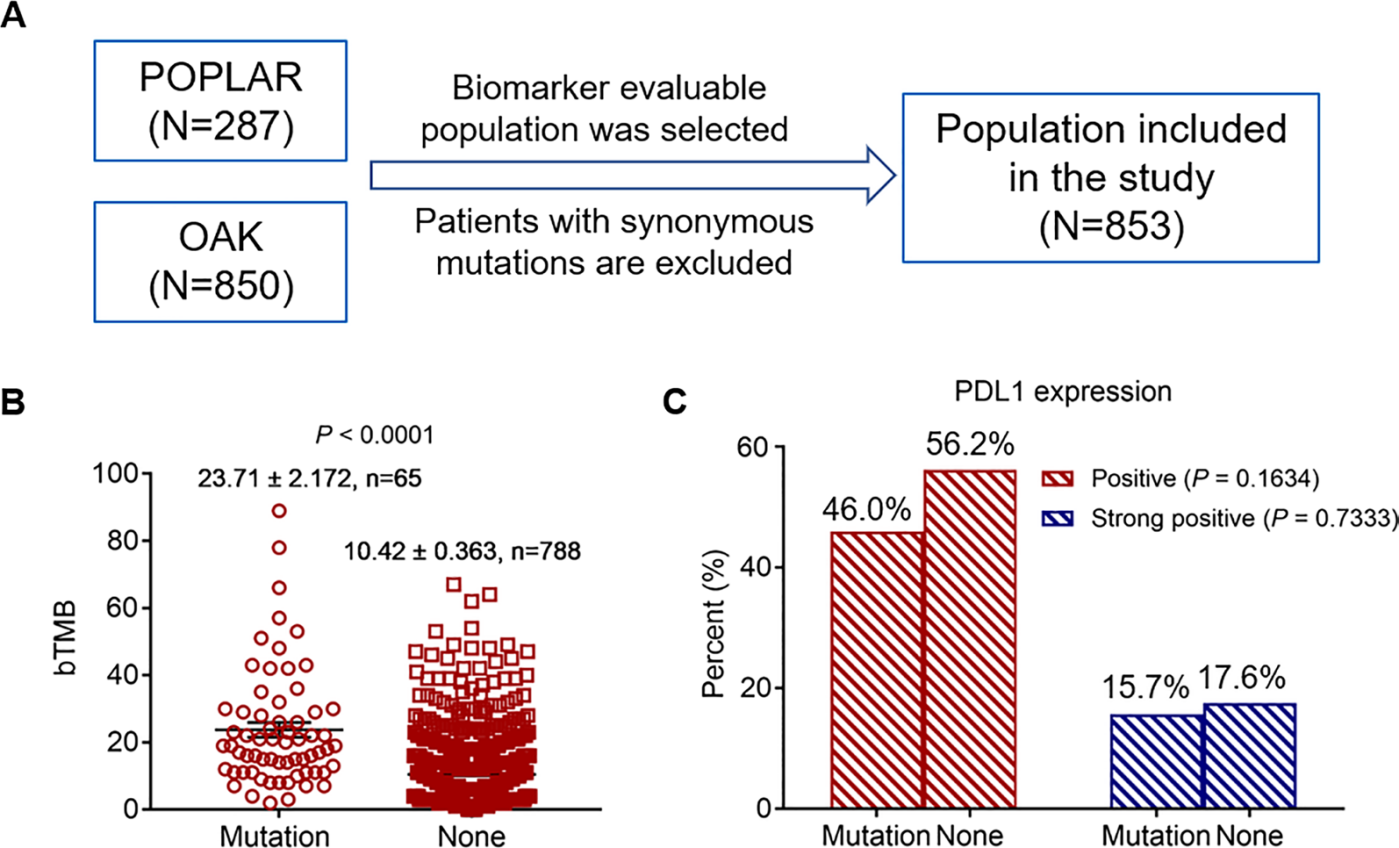
OAK and POPLAR cohort were used to evaluate association of bTMB and PD-L1 expression with EPHA5 mutation. This is a flow chart(**A**). Comparison of mutation count, TMB between EPHA5-mutated and wild-type tumors (*P* < 0.0001) (**B**). The difference of positive and strong positive PD-L1 expression between EPHA5-mutated and non. PD-L1 had higher proportion in non-mutant cohort compared with that in EPHA5-mutated one (56.2 vs. 46.0, *P* = 0.1634 and 17.6 vs. 15.7, *P* = 0.7333) (**C**). PD-L1: programmed death ligand 1; bTMB: blood tumor mutation burden

Considering that PD-L1 expression and TMB were associated with the response to ICIs in tumors, the correlations between mutations in EPHA5 and expression of PD-L1, and bTMB in both the mutant and wild-type cohorts were examined, as shown in Fig. 2. bTMB analysis showed that 65 patients had mutations in EPHA5, whereas 788 patients had wild-type EPHA5. The bTMB value showed a significant difference between the patients with mutant and wild-type EPHA5 (mean ± SD: 23.71 ± 2.172 vs*.* 10.42 ± 0.363, *P* < 0.0001, Fig. 2B). The analysis of PD-L1 expression showed that its positive rates in the patients with mutant and wild-type EPHA5 were 46.0% and 56.2%, respectively (*P* = 0.1634). The strong positive rates of PD-L1 expression were also analyzed between the two groups, showing no statistical difference (15.7% vs*.* 17.6%, *P* = 0.7333, Fig. 2C).

### Survival differences between the patients stratified by EPHA5 mutation status and treatment regimen

**Fig. 3:**
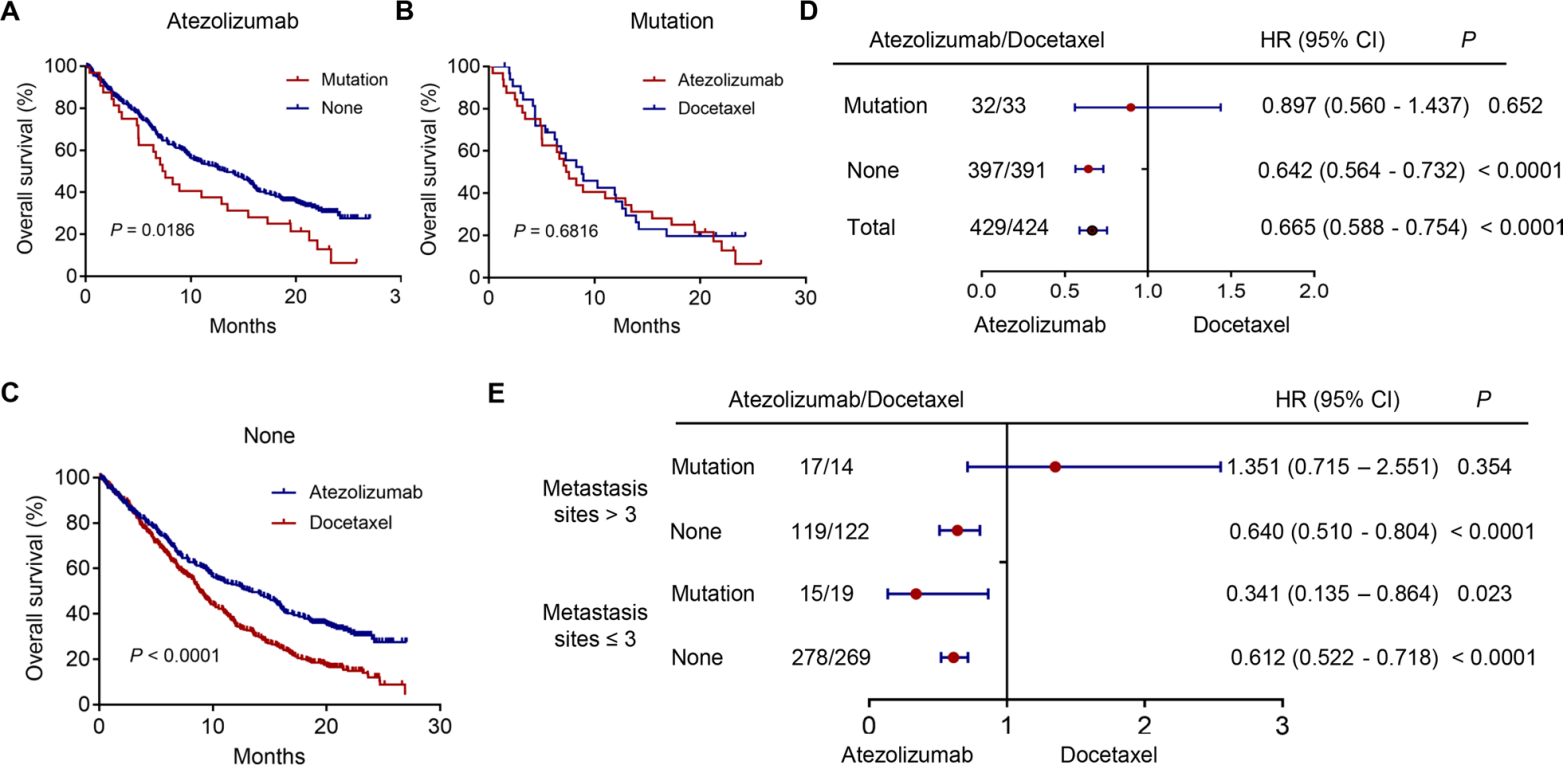
Survival analysis to evaluate the association between EPHA5 mutation and overall survival in patients in different treatment groups from OAK and POPLAR cohorts(*P* = 0.0186) (**A**). Survival difference between patients receiving atezolizumab and docetaxel stratified with EPHA5 mutation status (*P* = 0.6816 and *P* < 0.0001 respectively) (**B** and **C**). Subgroup survival of different variables including EPHA5 mutation status and metastatic sites number was plotted and presented on forest plot after multivariate Cox proportional hazard analysis (**D** and **E**). HR: hazard ratio

After treatment with atezolizumab, the survival rate of the patients with EPHA5 mutations was evaluated by performing a survival analysis. Kaplan–Meier analysis showed that the survival of the patients with EPHA5 mutation was worse as compared to that of the patients with wild-type EPHA5 (*P* = 0.0186), as shown in Fig. 3A. The differences in survival of patients, receiving atezolizumab and docetaxel, in the EPHA5 mutant cohorts were also compared. The results showed that atezolizumab had lesser beneficial effects on the patients with EPHA5 mutations as compared to those treated with docetaxel (*P* = 0.6816, Fig. 3B). However, among the patients with wild-type EPHA5, atezolizumab treatment could increase the OS as compared to docetaxel treatment (*P* < 0.0001, Fig. 3C). Kaplan–meier curves displayed in the supplementary file including four groups: EPHA5 mutation with metastases > 3, EPHA5 mutation with metastases ≤ 3, EPHA5 no mutation with metastases > 3, and EPHA5 no mutation with metastases ≤ 3 (Additional file 3: Fig. S1). The result shown that patients in group of EPHA5 no mutation with metastases ≤ 3 had longest survival than those in other groups (*P* = 0.0004).

### Survival difference between the patients receiving atezolizumab and docetaxel treatments based on several variables

Multivariate Cox regression analysis was performed to determine the independent predictors for the OS of patients, receiving atezolizumab treatment. Among all the patients and those with wild-type EPHA5, the patients, receiving atezolizumab treatment, showed better OS as compared to those, receiving docetaxel (HR = 0.665, 95% CI: 0.588–0.754, *P* < 0.001 and HR = 0.665, 95% CI 0.564–0.732, *P* < 0.0001), as shown in Fig. 3D. In the EPHA5 mutant subgroup, atezolizumab treatment could not improve the OS of patients as compared to that of the patients, receiving docetaxel (HR = 0.897, 95% CI: 0.56–1.437, *P* = 0.652).

Due to the correlations between EPHA5 mutations and metastatic sites number, the patients were further divided into two groups based on the number of metastatic sites. Among the patients having metastatic sites > 3, the patients with wild-type EPHA5 could benefit more from atezolizumab treatment as compared to docetaxel treatment. However, the patients having EPHA5 mutations could not benefit from atezolizumab treatment. Interestingly, among the patients having metastasis sites < 3, the survival benefit of atezolizumab treatment was observed in both the mutant and wild-type EPHA5 patients as compared to docetaxel treatment (HR = 0.341, 95% CI: 0.135–0.864, *P* = 0.023 and HR = 0.612, 95% CI: 0.512–0.718, *P* < 0.0001, Fig. 3E).

## Discussion

The OAK and POPLAR clinical trials showed that, among the previously treated NSCLC patients with high PD-L1 expression, the atezolizumab treatment could significantly improve the OS as compared to those treated with docetaxel [6, 7]. The improvement in OS strengthened with the increase in the expression level of PD-L1. The studies focused on the importance of assessing PD-L1 expression using immunohistochemistry as a predictive biomarker to select the patients for atezolizumab treatment as a second-line treatment. However, the atezolizumab treatment could not benefit some patients with high expressions of PD-L1. This might be due to the complexity of tumor immunity, which is involved multiple stepwise procedures [16, 17]. Moreover, besides the PD-L1 expression, TMB, TILs, and some genetic mutations, driving the composition of the tumor microenvironment, have been tested to predict their role in immunotherapy [18–20]. For example, due to the low expression of PD-L1 in EGFR-mutant advanced NSCLCs, the antI-PD-1/PD-L1 therapy had low efficiency, TMB, and infiltrating CD8 T cells as compared to wild-type ones [21].

This study investigated the rate mutations in EPHA5 and their role in predicting the efficacy of atezolizumab treatment in NSCLCs in the OAK and POPLAR cohorts. The role of EPHA family members in lung cancers has been reported in previous studies [22, 23]. The role of EPHA5 mutations has been less investigated in NSCLC patients, and studies are required to characterize their role in NSCLCs. In the current study, the somatic mutations, including missense and truncation, in the EPHA5 gene in the NSCLC patients were found in 7.6% (65/853) NSCLC patients in the OAK and POPLAR cohorts. The results showed that the patients with > 3 metastatic sites had mutations in the EPHA5 gene more frequently. These results indicated an addicted oncogenic gene in the subgroup of NSCLC patients [9, 24]. A recent basic study suggested that the mutations in EPHA5 could promote the migration and invasion of lung cancer cells by inhibiting the cytotoxicity of NK cells against cancer cells. This might help in explaining the results of the current study [10].

Evidence suggested that Eph receptors might mediate the activation of immune cells, thereby showing potential in selecting the ICIs. The correlations between EPHA5 mutations and factors, predicting the response of immunotherapy in NSCLCs, were also examined. The current study showed that EPHA5 mutations coexisted with those in TP53, STK11, and KEAP1 mutations. The missense mutations in TP53 were associated with the increased TMB and enriched in the NSCLC patients, who responded to ICIs [25, 26]. The mutations in STK11 might have an impact on the tumor microenvironment by promoting the neutrophils, which can suppress T-cells, and increasing the markers, which can exhaust T-cells; therefore, the STK11 mutations might result in an immunologically ‘cold’ microenvironment, conferring resistance to ICIs [25, 27]. The most frequently mutated genes in NSCLC include KEAP1; its mutations are associated with poor clinical outcomes among the patients treated with chemotherapy [28]. This gene mutation can also predict the worse outcomes in the NSCLC patients treated with ICI. However, more EGFR mutations were observed in the EPHA5-mutant group as compared to the patients' wild-type EPHA5. These results showed that EPHA5 could predict the efficacy of positive immune therapy. The two reported determinants, including PD-L1 expression and high levels of TMB, were correlated with immunotherapy response in the NSCLC patients. The current study showed no significant difference in the PD-L1 expression between the mutant and wild-type groups. However, higher levels of TMB were observed in the EPHA5 mutant group as compared to the EPHA5 wild-type group. These results demonstrated that the mutant EPHA5 could play a complex role in tumor immunity and might define a subpopulation of NSCLC patients, correlating with immune therapy response independent of TMB.

Based on these results, survival analysis was performed to confirm the correlations between EPHA5 mutations and OS of patients in the OAK and POPLAR cohorts. The results indicated that the patients with EPHA5 mutations could achieve relatively shorter OS as compared to those with wild-type EPHA5 among in the patients, receiving atezolizumab treatment. The patients having wild-type EPHA5 could benefit more from atezolizumab treatment as compared to docetaxel treatment. However, no difference was observed in the OS of EPHA5*-*mutant patients treated with atezolizumab and docetaxel. Multivariate analysis showed that the wild-type EPHA5 could independently predict more benefits for the patients treated with atezolizumab as compared to those treated with docetaxel. The patients with mutated EPHA5 exhibited a worse response to atezolizumab treatment, suggesting that EPHA5 mutation might be related to the tumor immune microenvironment. A recent study, conducted on the MSKCC immunotherapy cohort of lung adenocarcinoma patients, showed that the patients with mutations in EPHA5 could benefit more from immunotherapy as compared to those with wild-type EPHA5. However, these results were inconsistent with those of the current study. This might be due to the use of complex inhibits in the current study, such as anti-PD-1, anti-PD-L1, or anti-CTLA4 treatments, contributing to statistical bias. The current study had a uniform cohort, which might lead to more conclusive and reliable results. Moreover, in the present study, it was observed that the atezolizumab treatment benefitted the patients more with fewer metastatic sites as compared to those with more metastatic sites. The number of metastatic sites reflects TMB to some extent, which can predict the ICIs treatment response. For example, the high tumor metabolic tumor volume (tMTV) was correlated with the incidence of hyper-progressive disease, leading to a higher risk of adverse effects of ICIs; these results were similar to those observed in the present study [29].

Although the current study provided some interesting results, which might help in screening the NSCLC patients to receive more individualized treatment, there were some limitations to this study. First, the number of patients in the current study was small and included the patients from the OAK and POPLAR cohorts only, which might attenuate the statistical power of the analysis. Second, the data was insufficient to assess the correlations of EPHA5 mutations with immune cell infiltration, CNV counts, and related pathways to comprehensively elucidate the molecular mechanism for predicting the response of atezolizumab treatment. These results should be further verified using a large cohort.

## Conclusions

The current study showed that the NSCLC patients with EPHA5 mutations might represent a subpopulation and showed a worse response to atezolizumab treatment, thereby providing more accurate guidance for the NSCLC patients treated with atezolizumab (anti-PD-L1). A prospective study, having a larger sample size, should be performed to validate these results.

## Supplementary Information

**Additional file 1**: **Table S1**. Detailed information of EPHA5 mutations in NSCLCs from MSKCC cohort.

**Additional file 2**: **Table S2**. Detailed information of gene mutations in NSCLCs from OAK and POPLAR clinical trials.

**Additional file 3**: **Fig. S1**. Survival analysis divided in four groups: EPHA5 mutation with metastases > 3, EPHA5 mutation with metastases ≤ 3, EPHA5 no mutation with metastases > 3, and EPHA5 no mutation with metastases ≤ 3 (P = 0.0004).

## Data Availability

The datasets used during the current study available from the corresponding author on reasonable request.

## Abbreviations

NSCLC: Non-small cell lung cancer
PD-L1: Programmed death ligand 1
PD-1: Programmed death 1
EPHA5: Ephrin receptor5
TMB: Tumor mutation burden
TILs: Tumor infiltrating lymphomas
ICIs: Immune checkpoint inhibitors
MSKCC: Memorial sloan kettering cancer center
TP53: Tumor suppressor p53
KEAP1: Kelch-like ECH-associated protein 1
EGFR: Epidermal growth factor receptor
PTPRD: Protein tyrosine phosphatase, receptor type D
PTPRT: Protein tyrosine phosphatase receptor T
NF1: Neurofibromatosis type 1
FAT1: FAT atypical cadherin 1
HGF: Hepatocyte growth factor
bTMB: Blood TMB
tMTV: Tumor metabolic tumor volume
HR: Hazard ratio
CI: Confidence interval
