# Supplementary material for: EPHA5 mutation was associated with adverse outcome of atezolizumab treatment in late-stage non-small cell lung cancers
Source: BMC Pulm Med. 2022 Sep 19;22:356. doi: 10.1186/s12890-022-02161-1 (PMC9487080; doi:10.1186/s12890-022-02161-1)
Supplement: Supplementary file 3 — Additional file 3: Fig. S1. Survival analysis divided in four groups: EPHA5 mutation with metastases > 3, EPHA5 mutation with metastases ≤ 3, EPHA5 no mutation with metastases > 3, and EPHA5 no mutation with metastases ≤ 3 (P = 0.0004). [file 12890_2022_2161_MOESM3_ESM.docx]

Figure1s Survival analysis divided in four groups according to metastases number and EPHA5 mutation status.


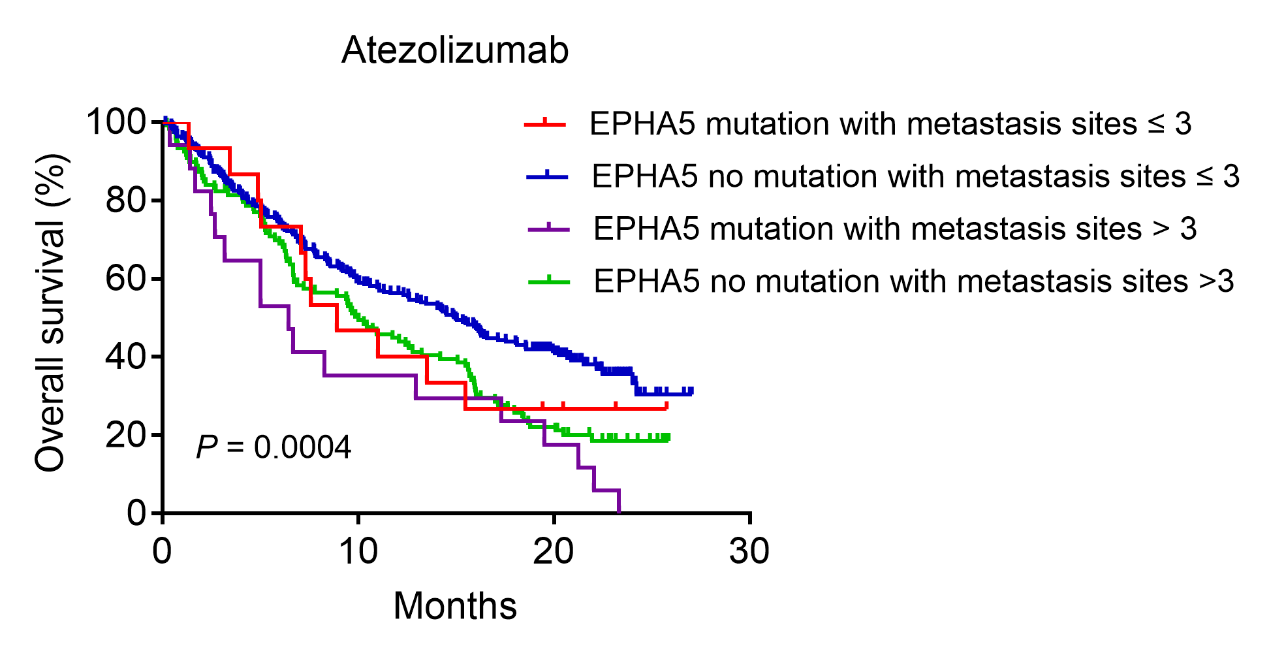


The figure showed that there were four groups: EPHA5 mutation with metastases >3, EPHA5 mutation with metastases ≤3, EPHA5 no mutation with metastases >3, and EPHA5 no mutation with metastases ≤3. Patients in group of EPHA5 no mutation with metastasis sites ≤3 had longest overall survival compared to other three groups (*P*=0.0004).
